# The Influence of pH on the Scleroglucan and Scleroglucan/Borax Systems

**DOI:** 10.3390/molecules22030435

**Published:** 2017-03-09

**Authors:** Claudia Mazzuca, Gianfranco Bocchinfuso, Antonio Palleschi, Paolo Conflitti, Mario Grassi, Chiara Di Meo, Franco Alhaique, Tommasina Coviello

**Affiliations:** 1 Department of Chemical Sciences and Technologies, University of Rome “Tor Vergata”, Via della Ricerca Scientifica, 00133 Rome, Italy; claudia.mazzuca@uniroma2.it (C.M.); gianfranco.bocchinfuso@uniroma2.it (G.B.); antonio.palleschi@uniroma2.it (A.P.); paolo.conflitti@uniroma2.it (P.C.); 2 Department of Engineering and Architecture, University of Trieste, P.le Europa 1, 34127 Trieste, Italy; mario.grassi@di3.units.it; 3 Department of Drug Chemistry and Technologies, “Sapienza”, University of Rome, P.le Aldo Moro 5, 00185 Rome, Italy; chiara.dimeo@uniroma1.it (C.D.M.); franco.alhaique@uniroma1.it (F.A.)

**Keywords:** scleroglucan, rheology, swelling, Congo red, molecular dynamics simulations, principal component analysis

## Abstract

The effects that an increase of environmental pH has on the triple helix of scleroglucan (Sclg) and on the Sclg/borax hydrogel are reported. Rheological experiments show that the hydrogel is less sensitive to pH increase than Sclg alone, while at pH = 14 a dramatic viscosity decrease takes place for both systems. This effect is evidenced also by the reduced water uptake and anisotropic elongation detected, at pH = 14, by the swelling behaviour of tablets prepared with the Sclg/borax system. On the opposite, a different behaviour was observed with guar gum and locust bean gum tablets, tested as reference polysaccharides. The effect of pH on the structure of Sclg and Sclg/borax was investigated also by means of spectroscopic approaches based on the interaction between Congo red (CR) and the Sclg triple helix. Obtained results indicated that the CR absorbance maximum is shifted as a function of pH and by the presence of borax. Principal component analysis allowed very precise identification of the pH value at which the Sclg helix collapses. Molecular dynamics simulations of the Sclg/borax–CR complex indicated that, at physiological pH, only a few ordered configurations are populated, according to the induced circular dichroism (CD) spectrum evidence.

## 1. Introduction

**Scheme 1:**
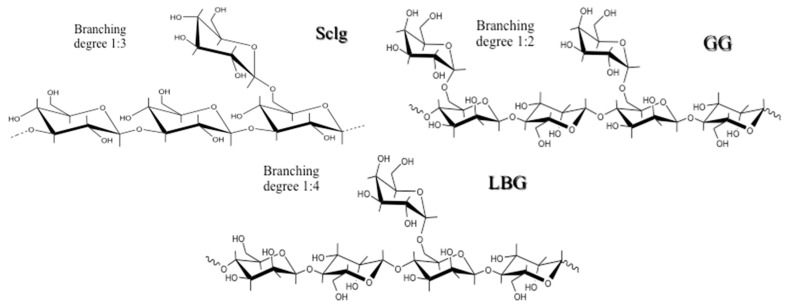
Repeating units of scleroglucan (Sclg); guar gum (GG); and locust bean gum (LBG).

Scleroglucan (Sclg) is a natural polysaccharide produced by fungi of the genus *Sclerotium*, extensively studied for various commercial applications (secondary oil recovery, ceramic glazes, food, paints, cosmetics, etc.) and also for its interesting pharmaceutical applications [1,2,3]. The polymer exhibits a backbone build up by (1→3) linked β-d-glucopyranose units with single glucopyranose side chains linked β-(1→6) (Scheme 1). From a structural point of view, Sclg dissolves in water with a triple-helix conformation sustained by a network of interchain H-bonds among the hydroxyl groups linked to the C-2 atoms of the glucose units of the backbone. This H-bond network is responsible for the high stiffness shown by the polymer.

Furthermore, Sclg, just like other polysaccharides [4], is capable of interacting with borax, leading to complex systems in which chemical bonds and electrostatic interactions are both involved [5]. In particular, in the case of Sclg, the presence of borax allows the formation of domains where the interactions among several triple helices (triplexes) of the polysaccharide lead to a hydrogel network with specific and peculiar properties [6] such as a remarkable anisotropic swelling of tablets, prepared from the freeze-dried hydrogel [7]. This effect was interpreted as the consequence of the particular structure present at a microscopic level, enhanced by the compression force applied during tablet preparation. The presence, within the network, of *soft* nanochannels capable of playing a fundamental role in this unusual behaviour was suggested by means of molecular dynamics (MD) simulations [8]. Furthermore, NMR studies evidenced a mechanism of hyperdiffusion of water molecules along one direction, thus supporting the hypothesis of the presence of well-ordered nanostructures [9]. These results provided appropriate explanations for the behaviour of Sclg in the various applications that have been reported, especially in the field of pharmaceutics [1]. Taking into account that the Sclg triple helix breaks down at pH higher than 13, the aim of the present study was to investigate the effect that an increase of pH may have on the overall structure of the Sclg/borax system. In particular, the effect of pH = 14 on the anisotropic swelling of Sclg/borax tablets was tested. For this purpose, two approaches were followed: in one case, the tablets were prepared from the polymer dissolved in an alkaline solution (pH = 14) and then swelled in distilled water; in the other case, the polymer was first dissolved in water and the prepared tablets were then swelled in the alkaline solution (pH = 14). Taking into account that at pH 14 the triple helix of Sclg is no longer present [10,11,12], for an appropriate comparison, tablets with guar gum (GG) and locust bean gum (LBG) (Scheme 1) were also prepared and tested in the same environmental conditions. GG and LBG are neutral, water-soluble galactomannans consisting of a linear backbone of β(1→4)-linked d-mannopyranose units with the presence of randomly attached α(1→6)-linked galactopyranose units as side chains. GG and LBG, unlike Sclg, dissolve in aqueous solution as random coils. These two polysaccharides were chosen because, according to previous results, GG/borax and LBG/borax tablets also showed an anisotropic swelling when soaked in distilled water [13,14]. By means of rheological measurements, the influence of pH on flow curves and mechanical spectra of Sclg/borax was also evaluated. For a deeper insight about the effect of pH on the structure of the Sclg/borax complex, Congo red (CR)—a well-known probe capable of interacting with biomacromolecules, including polysaccharides [15,16]—was used for a spectroscopic characterization (UV and circular dichroism (CD)). In particular, CR is useful for following structural transitions in macromolecules (in our case, Sclg triple-helix denaturation) because changes in environmental conditions induce a shift in the maximum of its absorption spectrum [17]. Principal component analysis (PCA), allowed a clear distinction to be made between the CR spectra in the presence of Sclg alone and Sclg/borax, and it was possible to identify the pH values at which the triple helix collapses. Although the abovementioned techniques are suitable for the characterization of polysaccharides, they are unable to provide a structural picture of the systems. For this purpose, a combined approach of experimental and computational data can be particularly useful. Thus, in the present paper, MD simulations allowed the acquisition of a detailed description about the structure of the complex that is formed between CR and Sclg/borax.

## 2. Results and Discussion

### 2.1. Rheology

**Figure 1:**
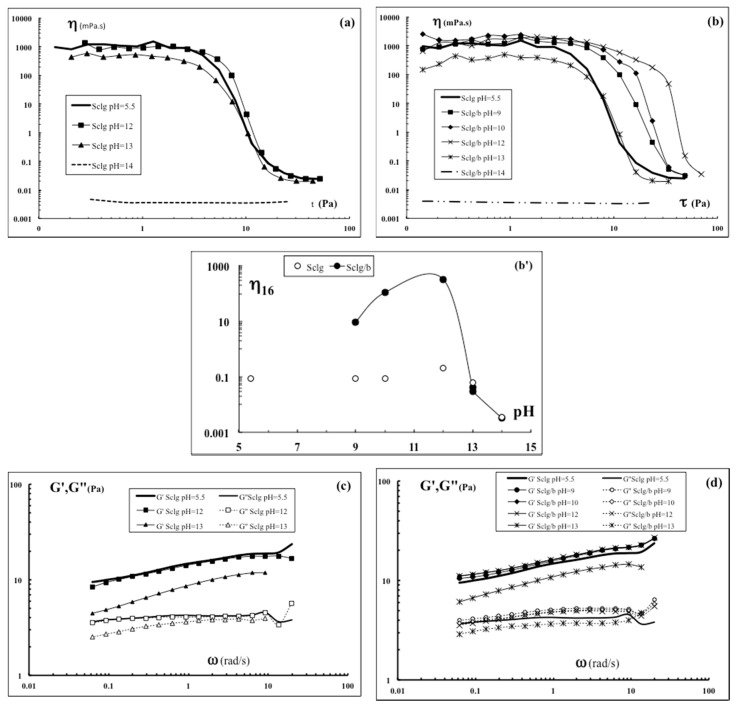

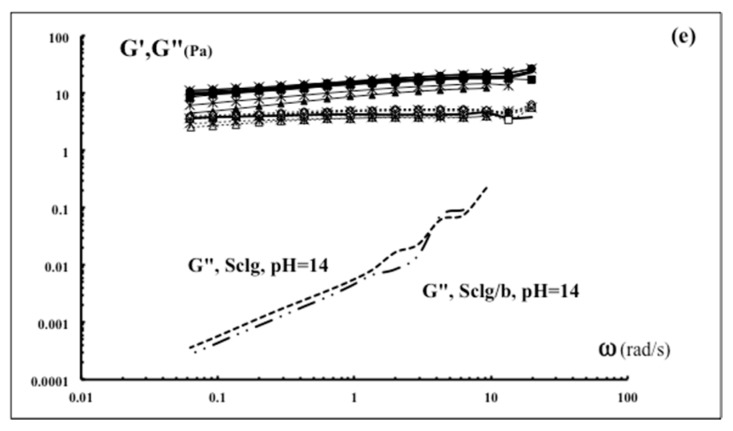
Flow curves of Sclg (**a**) and Sclg/borax (**b**) samples at different pH values; (**b’**): viscosity at stress τ = 16 Pa (η_16_) for Sclg and Sclg/borax samples at different pH values. Mechanical spectra of Sclg (**c**) and Sclg/borax (**d**) at different pH values. In Figure (**e**), the data for both samples are reported together with the loss modulus G” values recorded at pH = 14 (polymer concentration, cp = 0.7%, T = 25 °C). In Figure (**b**–**e**), for an appropriate comparison, the data of Sclg are also shown.

Figure 1a,b indicates that all the studied systems, with the exception of those carried out at pH = 14, exhibit the same general characteristics, as far as the shear viscosity trend is concerned. Indeed, all of them show the typical shear-thinning behaviour where the upper Newtonian plateau (around 1000 Pa·s) is connected to the lower Newtonian plateau (around 0.03 Pa·s) by an almost linear trend (in logarithmic scale). However, despite this general similarity, the systems differ for the stress τ at which the viscosity breakdown occurs. For example, for Sclg solutions, up to pH = 13, viscosity breakdown occurs around 9 Pa, while at pH = 14 no breakdown is detectable as the system is essentially Newtonian with a viscosity approximately 4 times that of water at 25 °C (1 m·Pa·s). Actually, at such high pH values, the integrity of the polymeric network is seriously compromised due to the Sclg triple-helix collapse [18] and to chemical degradation of the chains.

The addition of borax does not significantly affect the upper and lower Newtonian plateau, but it shifts forward the viscosity breakdown up to approximately 20–30 Pa. This suggests that borax promotes the formation of linkages among Sclg chains, leading to networks more resistant to deformations. When pH is raised to 14, the Sclg–borax solution again acquires the Newtonian behaviour with a viscosity close to that of Sclg solutions. This further confirms the damages induced by high pH values to the Sclg chains (breakdown of the triple helices and hydrolysis of the glycosidic linkages). Figure 1b’ sums up the effects of pH on flow properties related to the systems with and without borax. Indeed, Figure 1b’ reports the pH dependence of the system viscosities evaluated at τ = 16 Pa (η_16_: this value corresponds to the beginning of the second Newtonian plateau in Figure 1a). While in the case of borax-free systems, regardless of pH, η_16_ is almost constant and less than 0.1 Pa·s, in the case of Sclg/borax systems, η_16_ increases up to pH 12, then at pH = 14 it abruptly falls down below 0.01 Pa·s. This result further supports the different structures between the borax free and the Sclg/borax systems. Also, stress sweep tests (see Table 1) support the results obtained from the flow curve experiments. All systems, regardless of the presence of borax, and at pH lower than 14, show the typical weak gel behaviour, where the storage modulus *G*’ is clearly higher than the loss modulus *G*″ (*G*’ is about 3 times *G*″) and both moduli are constant with the applied stress up to the linear viscoelastic threshold. On the other side, when pH = 14, only *G*″ is detectable, indicating the typical behaviour of a solution. This means that, due to pH, the breakage of Sclg chains leads to the destruction of the polymeric network. In order to discriminate the effect of pH and borax addition on the weak gel systems, Table 1 reports, for the different systems, the limit of the linear viscoelastic field in terms of critical parameters (i.e., the values of the critical storage (*G*’_c_) and loss (*G*″_c_) moduli and the values of the critical stress (τ_c_) and deformation (γ_c_)). It can be observed that Sclg critical parameters are almost unaffected by pH up to the value of 12, and only a small reduction of critical parameters *G*’_c_, *G*″_c_, and τ_c_ occurs at pH = 13 (while γ_c_ remains almost constant), due to the connectivity loss of the polymeric network. The addition of borax increases the gel strength and, up to pH 13, *G*’_c_, *G*″_c_, and τ_c_ are higher in comparison to those of the samples without borax. At the same time, as in the case of the Sclg systems, when pH 13 is reached, a reduction of *G*’_c_, *G*″_c_, and τ_c_ occurs and the further increase of pH up to 14 leads the complete breakdown of the three-dimensional polymeric network, also related to the chemical degradation of the chains.

Frequency sweep tests (Figure 1e) confirm that, at pH = 14, regardless of the presence of borax, the storage modulus cannot be detected and only the loss modulus appears due to the breakage of the three-dimensional polymeric network. Additionally, at pH < 14, frequency sweep tests (Figure 1c,d) confirm the weak gel nature of Sclg and Sclg–borax systems, as *G*’ is always 2–3 times *G*″ and both of them are slightly dependent on angular frequency ω. Moreover, it can be noted that the addition of borax does not lead to a significant increase of *G*’ and that, up to pH = 12, all the systems (with and without borax) show very similar behaviours. The mechanical spectra seem to be, at a first glance, in contrast with the flow curves and the stress sweep test, where the effect of borax was always detectable and well evident. However, the overall results from the rheological tests can be explained by taking into account that:

(i) the presence of borax affects the polymeric network architecture mainly at long range, the condition actually tested by flow curve and stress sweep experiments, carried out also beyond the linear viscoelastic threshold;
(ii) in the frequency sweep experiments, the systems are explored only at short range, with small deformations imposed in the linear viscoelastic range, thus less affected by the presence of borax.

Finally, at pH = 13, (Figure 1c,d), although both systems (with and without borax) show *G*’ values higher than those of *G*″, they undergo a clear decrease in comparison to the samples at pH < 13. Furthermore, the greater frequency dependence that is evident for the storage moduli clearly indicates the shifting of the systems towards a sol condition.

### 2.2. Water Uptake and Dimensional Increase Studies

It has been reported that tablets, prepared with the Sclg/borax and GG/borax freeze-dried hydrogels, when soaked in aqueous media, undergo an anomalous swelling, essentially along one direction. The same behaviour was observed in the case of LBG, although to a much lesser extent [6,7,8,9,13,14,19,20]. In order to test the possible effect of high pH values on the swelling behaviour of Sclg/borax tablets, two different types of experiments were carried out:

(i) Sclg was first dissolved at pH = 14 before the addition of borax and the tablet preparation. The swelling experiments were then carried out in distilled water (at 37 °C).
(ii) Sclg was first dissolved in distilled water before the addition of borax and the tablet preparation.

The swelling experiments were then carried out in aqueous solutions at pH = 14 (at 37 °C).

For an appropriate comparison, the same tests were performed using tablets obtained with GG/borax and LBG/borax following the same procedures above reported.

**Figure 2:**
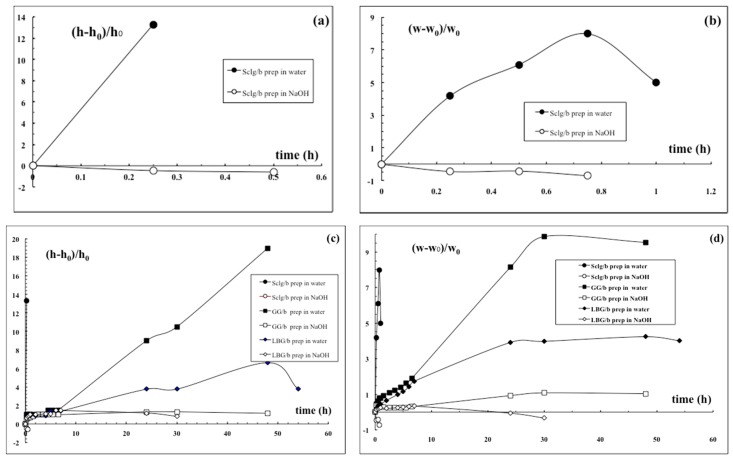
Relative increase of tablet heights for Sclg/borax (**a**); Sclg/borax, GG/borax, and LBG/borax (**c**); tablet water uptake for Sclg/borax (**b**); Sclg/borax, GG/borax, and LBG/borax (**d**). (Full symbols: tablets prepared in water and swelled at pH = 14; empty symbols: tablets prepared at pH = 14 and swelled in water. T = 37 °C).

**Figure 3:**
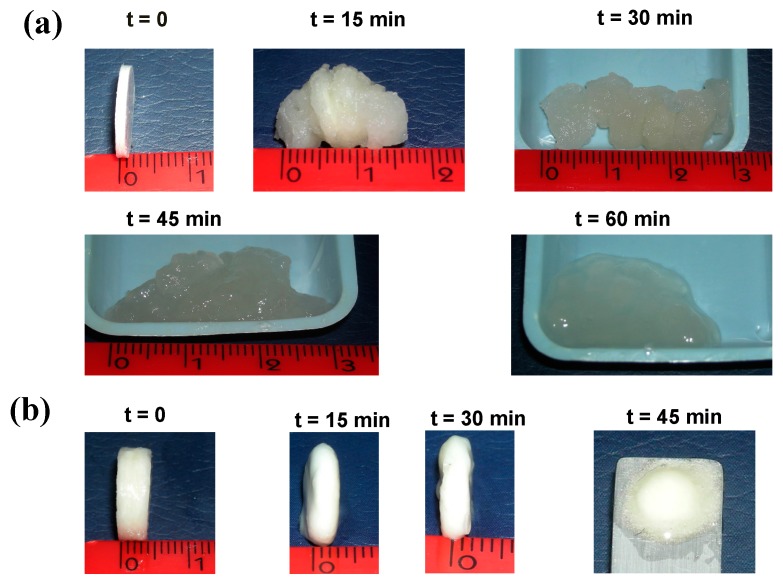
Pictures of Sclg/borax tablets, prepared in water and swelled in NaOH (pH = 14) (**a**) or prepared in NaOH (pH = 14) and swelled in water (**b**), after swelling, for different periods of time, at 37 °C.

The results, reported in Figure 2, clearly show an interesting behaviour, which differs for the three kinds of tablets. First of all, it must be pointed out that, in all cases, the presence of NaOH reduced significantly both water uptake and elongation in comparison to the data obtained when only distilled water was used for tablet preparations and swelling experiments (see Figure 3 in Reference [13]). In fact, when the polymers were dissolved at high pH values (preparation i) the hydrolysis of glycosidic bonds led to a simultaneous drastic reduction in molecular weights; consequently, the tablets, when soaked in water, showed only a slight water uptake and a slight anisotropic elongation. In the case of Sclg, a mass decrease had already occurred after 15 min, and for this reason an “apparent negative elongation” was recorded. Actually, the high pH values promote a depolymerisation reaction but also induce a conformational transition helix–coil, thus destabilising the original very stable triple-helix conformation. Also in the case of the two tested galactomannans the effect was evident, although to a lesser extent than that obtained with Sclg. In fact, it is known that the anisotropic elongation is due to the borax interaction with the glucose side chains of the polymers, which leads to an alignment of the macromolecules. As a consequence, LBG—because of the lower number of side chains (branching degree 1:4), when compared with GG (branching degree 1:2)—always shows a more evident reduction of the effects induced by the borax, as reported in Figure 2c,d. Furthermore, due to the dissolution of the tablets, for GG and LBG it was possible to carry out the experiments for a maximum of 1 or 2 days. A quite different behaviour was observed when the polymers were dissolved in water and the tablets were then soaked in NaOH aqueous solutions (pH = 14) (preparation ii). In the case of Sclg, the effect was dramatic and very rapid: the weight loss was already recorded after 45 min. In the case of GG and LBG, the hydrolysis reaction was rather slower. The hydrolysis occurred in a heterogeneous system due to the presence, at the beginning, of the polymeric chains as a solid phase (tablets). Consequently, the weight loss was appreciated after only 1 day.

These peculiar differences observed between Sclg (from one side) and GG and LBG (from the other side) have to be ascribed to different conformations of the three polymers. Sclg is a triple helix and the effect of NaOH is revealed to be extremely rapid and dramatic in terms of chain rigidity and tablet anisotropic elongation. On the other side, GG and LBG, which are flexible chains, in the presence of borax show self-healing effects [21,22] that counterbalance, at least partially, the hydrolysis induced by NaOH. The effect of NaOH is particularly evident when the swelling data at pH 14 are compared with those obtained in distilled water (see Figure 3 in Reference [13]). Actually, the interaction of Sclg from one side and GG and LBG from the other side with borax takes place in a different way: in the case of Sclg, the borax promotes mixed (chemical and physical) interactions between the triple helices (at least for the samples prepared in distilled water) while, in the case of GG and LBG, the borax forms chemical bridges between chains by means of linkages that are reversible. During the swelling in NaOH, the labile nature of the borax cross-links in the GG, and to a lesser extent in the LBG, making the interchain interactions able to undergo the needed rearrangement in order to keep, for a while, the tendency to give the anisotropic elongation of the tablets. To better illustrate the data reported in Figure 2, the pictures of Sclg/borax tablets prepared in distilled water (a) or in NaOH (b), and swelled in NaOH (a) or in distilled water (b), for different periods of time, are shown in Figure 3.

### 2.3. Spectroscopic and Chemometric Analysis

**Figure 4:**
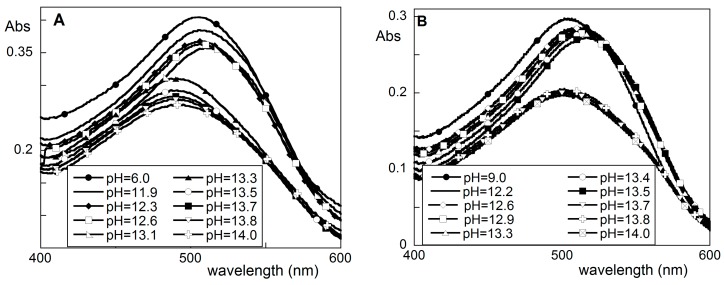
Absorbance spectra of Congo red (CR) in water solution of Sclg (**A**); and Sclg/borax (**B**) at different pH values.

The different behaviours of the Sclg and Sclg/borax solutions as a function of pH was examined also by characterising the CR–polysaccharide interaction using UV–vis absorption and CD spectroscopy. CR is a probe widely used to follow conformational transitions in biomacromolecules, including polysaccharides [15,17,23,24,25]. Changes in the conditions surrounding the macromolecules (i.e., the interaction of the tested polymer with other molecules) can induce a well-detectable shift in the maximum of the CR absorption spectra. As reported in Figure 4, the absorption maximum of CR in solution was red-shifted in the presence of Sclg by increasing pH up to 13.1; at higher pH values, the absorption maxima decrease abruptly at almost constant values. This behaviour correlates well with the known Sclg transition, which occurs roughly at pH 13 from an ordered (triple helix) to a single-chain conformation, thus indicating that CR is a useful probe to follow this structural rearrangement. A very similar behaviour was observed with the Sclg/borax complex. However, in this case, the transition pH occurred at pH 13.5 (Figure 4) because of the stabilising effect of borax on the triple-helix structure [25].

**Figure 5:**
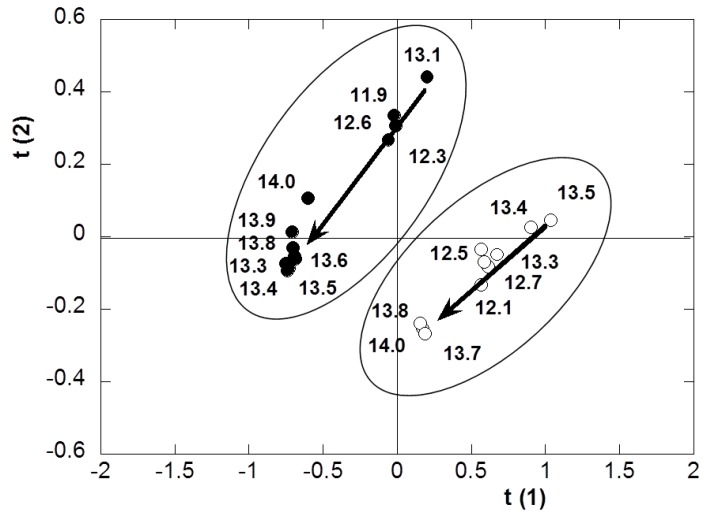
Projection of the Sclg (filled circles) and Sclg/borax (empty circles) absorption spectra onto the space spanned by the first two principal components (PCs). The arrows indicate the transition observed in the spectra at pH = 13.1 (Sclg) and pH = 13.5 (Sclg/borax).

The result of PCA on the absorption spectra is reported in Figure 5. The analysed experimental data are projected in the space of the first two principal components that take into account more than 99% of the original variance. A clear distinction between the CR spectra in Sclg and Sclg/borax solution samples can be observed: the complexation of the polysaccharide with borax has a strong influence on the CR binding, affecting both the first (that accounts for the 90% of the variability) and the second principal component (that accounts for the 9% of the variability). Very important is the variation of pH that has a remarkable effect on the absorption spectra: the position in the PCs space of the Sclg and the Sclg/borax samples undergoes a remarkable shift for pH higher than 13.1 and 13.5, respectively. This shift is related to the Sclg transition from triple helix to single strand. It must be also pointed out that at higher pH values, further changes occur in the absence of borax, while such changes are not detectable in the case of Sclg/borax samples, thus suggesting that the stabilising effects of the borax still remain in the Sclg single strand.

**Figure 6:**
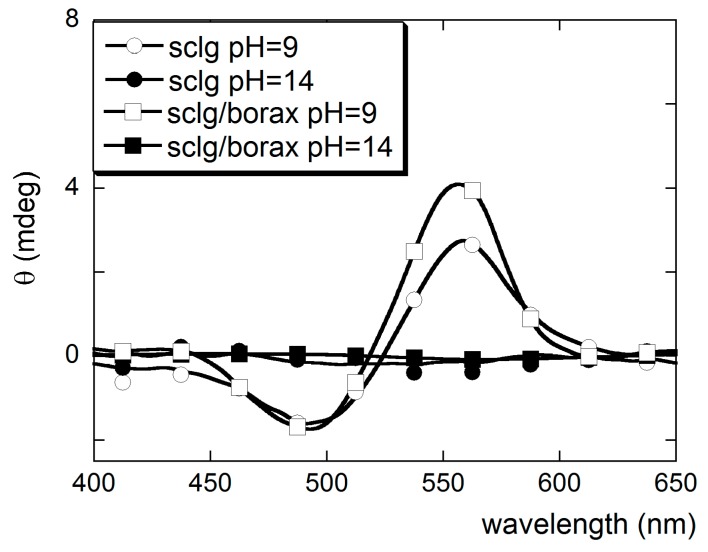
Circular dichroism (CD) spectra of CR–Sclg complex (circles) and CR–Sclg/borax (squares), at pH = 9 (empty symbols) and 14 (filled symbols) in water solution.

The CD spectra (Figure 6), corresponding to the absorption band of CR, were acquired at pH 9 and 14. At pH 9, a well-defined band is present in both the Sclg and Sclg/borax samples. For the Sclg/borax sample a higher induced CD signal is registered, due the presence of borax, which affects the mobility of the lateral domains of Sclg chains [8].

In all samples, with and without borax, the CD signal is lost at pH 14. These data confirm that, at pH 14, a structural transition between an ordered structure (triple helix) and a single chain takes place for both systems.

### 2.4. Molecular Dynamics (MD) Simulations

**Figure 7:**
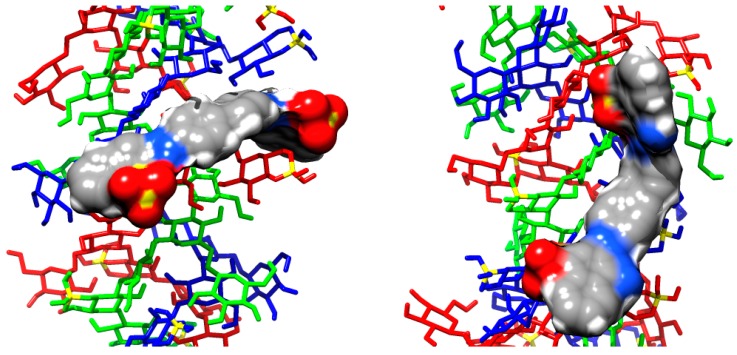
Structures of the complexes sampled during the molecular dynamics (MD) simulations of the triple helix in the presence of CR. The two structures are the more representative of the complexes with perpendicular (on the left) and parallel (on the right) orientation of CR with respect to the triple-helix axis. Sclg is reported with a stick representation, the three strands are coloured in red, blue, and green, respectively. The boron atom is evidenced in yellow. The CR molecules are reported as a surface, coloured by elements.

The interactions between the Sclg/borax and CR were investigated by means of MD simulations. Six independent simulations were carried out by starting from random positions of CR with respect to the triple helix and, in all cases, at a starting distance greater than 1.6 nm. The interaction between CR and triple helix takes place in different ways, as the triple helix has not a single binding site for CR (a different situation occurs for proteins that have only a specific binding site for their ligands). This situation does not allow a straightforward cluster analysis to identify the most representative structures from the six independent simulations. A preliminary separation of the sampled complexes has been carried out by analysing the angle between the principal axis of triple helix and CR, leading to two main representative families of structures in which CR lays in a parallel or perpendicular position with respect to the triple helix axis. Two examples, representative of these two families, are reported in Figure 7.

These structures were obtained by performing two independent cluster analyses on the two families above described. It should be pointed out that the configurations belonging to each one of the two families (i.e., with CR perpendicular and parallel) are actually very similar. These data support the spectroscopic evidence that an induced CD on CR can be observed as a consequence of the interaction with Sclg triple helix. On the other side, a different behaviour was obtained when the complexes between CR and Sclg single strand (i.e., Sclg conformation at pH 14) were studied [25] and no induced CD could be detected. These simulations do not allow evaluating which one of these two complexes is more stable. Anyhow, it can be asserted that, once formed, both complexes were very stable. In the configuration with a perpendicular orientation of CR, a tighter interaction between the probe and Sclg/borax takes place, and CR intercalates in a groove of the triple helix. It has to be underlined that these structures are similar to those observed in the case of the simulation of Sclg (without borax) and CR [25]. These evidences further support the above-reported statement that the presence of borax does not significantly influence the CR–Sclg interaction.

## 3. Materials and Methods

### 3.1. Materials

Sclg (Actigum CS 11) was provided by Cargill (Minneapolis, MN, USA); GG and LBG by CarboMer, Inc. (San Diego, CA, USA); borax and NaOH Normex were Carlo Erba (Milano, Italy) products; and Congo Red (CR) a Sigma Aldrich (St. Louis, MO, USA) product. For the sample preparations, distilled water was always used.

### 3.2. Methods

#### 3.2.1. Polysaccharide Purifications

A given amount of each polymer was dissolved in distilled water (polymer concentration, cp = 0.5% *w*/*v*), and then kept under magnetic and mechanical stirring at room temperature for 24 h. In the case of GG and LBG samples, the dissolution step was carried out at 60 °C. The obtained solutions were exhaustively dialysed at 7 °C against distilled water and then freeze-dried. The molecular weight cutoff of the dialysis tubing was 12,000–14,000.

#### 3.2.2. Hydrogel and Tablet Preparation

For the hydrogel preparation, an appropriate amount of each polymer (i.e., Sclg, GG, and LBG) was magnetically stirred in water for 24 h (pH of the solutions = 5.5). The calculated amount (i.e., moles of borax = moles of repeating units of polymer) of 0.1 M borax solution was then added and the system was left under magnetic stirring for 5 min (pH of the solutions = 9.0 because of the borax buffer effect). The obtained samples (cp = 0.7%, *w*/*v*) were kept overnight at 7 °C for gel-setting. For the preparation of the tablets, 200 mg of each polymer was used, and after the gel-setting the samples were freeze-dried. Tablets were prepared from the freeze-dried samples with an IR die (PerkinElmer hydraulic press) using a force of 5.0 kN for 30 s. The weight of tablets was 230 ± 10 mg. The diameter and the thickness, measured with a screw gauge, were 13.0 ± 0.1 mm and 1.05 ± 0.02 mm, respectively. Tablets were also prepared starting from pH = 14 aqueous solutions of the polymers.

#### 3.2.3. Water Uptake and Dimensional Increase Studies

The swelling of Sclg/borax, GG/borax, and LBG/borax tablets, prepared from distilled water solutions, was carried out by soaking the tablets in NaOH at pH = 14 at 37 °C. On the other side, the swelling of tablets, prepared from the alkaline polymer solutions (pH = 14) was carried out by soaking the tablets in distilled water at 37 °C. At fixed time intervals, the tablets were withdrawn, the excess of solvent was removed with soft filter paper for 5 s, and then the corresponding weights and dimensional variations along the longitudinal axis were determined by means of a screw gauge with an accuracy of ±0.1 mm. No remarkable variations of cross-section dimensions were detected during the swelling process. All experiments were carried out in triplicate and the obtained values always lay within 10% of the mean.

#### 3.2.4. Rheology

A rheological characterisation of the polymer solutions and of the gels prepared at different pH values was performed, at 25 °C, by means of a controlled stress rheometer Haake Rheo-Stress RS150, using, as a measuring device, a shagreened plate and plate apparatus (PP35 TI: diameter = 35 mm; gap between plates = 1 mm). This kind of device is needed to avoid possible slippage phenomena at the wall [26]. To avoid gel shrinking, due to a possible solvent evaporation, the equipment was kept inside a glass bell with a constant moisture level. Rheological properties were studied under small and large deformations, as well as in flow conditions, by applying different procedures: stress (ν = 1 Hz, ω = 2πν = 6.28 rad/s) and frequency (in the linear viscoelastic region; constant deformation γ = 0.05) sweep, and flow curves (maximum waiting time, 45 s, for the determination of shear viscosity at each stress).

#### 3.2.5. Conformational Transition Studies

The capability of polysaccharides to adopt the helix conformation was examined by characterizing the CR–polymer complexes [15,23]. Aliquots of a NaOH stock solution were added to the polymer samples (1 g/L) containing 10 µM CR, in order to obtain the desired pH values. The solutions were left to equilibrate until their UV–vis or circular dichroism (CD) spectra did not change with time (about 30 min). As a reference, similar experiments were carried out on a 10 µM CR solution. The absorption spectra were recorded with a Cary 100 spectrophotometer (Varian, Palo alto, CA, USA) while CD spectra were performed on a J-600 apparatus (Jasco, Easton, MD, USA). In both cases, a 1 cm path length quartz cuvette was used. For these experiments, 1 g/L of Sclg solution, with or without borax in a 1:1 ratio was used. Results were compared to those obtained with GG and LBG solutions (1 g/L).

#### 3.2.6. Molecular Dynamics Simulation

MD simulations were carried out using the software package Gromacs v4.6.5 [27]. The force field parameters for Sclg and borax were those previously used [6,8,28], while for CR, the parameters were taken from Król et al. [29]. The simulations were carried out on one triple helix, composed of chains of 7 Sclg repeating units, 1 CR molecule, and roughly 17,000 water molecules [30]. These systems were simulated in a cubic box (8.2 nm for each side) in an isothermal–isobaric (NPT) ensemble. Electrostatic interactions were calculated with the particle mesh Ewald (PME) algorithm [31] (cutoff 1.4 nm); sodium ions were added to assure electroneutrality conditions. A double cutoff was used for the van der Waals interactions (1.0–1.4 nm). Pressure and temperature were kept constant by the Berendsen algorithm [32]. Six independent starting configurations were prepared with CR at different random distances (higher than 1.6 nm) and orientations with respect to the polysaccharide. After the interactions between CR and triple helix reached equilibrium, the trajectories of the systems were then analysed for 20 ns. The clusterisations were obtained using a homemade script, to evaluate the angle between CR and the triple helix, and the g_cluster tool of the GROMACS software package with a cutoff of 0.3 nm. G_cluster was used also to obtain the more representative structure in the clusters (i.e., the structure with the smallest average root-mean-square deviation from all the other structures of the cluster). The structural images were obtained using the Chimera software [33].

#### 3.2.7. Chemometric Analysis

The results of UV–vis experiments on the binding of CR with polysaccharides were processed by means of the chemometric exploratory data analysis technique, principal component analysis (PCA) [34,35]. Using PCA, a data set is compressed by projecting the experimental data on a low-dimensional space without losing the relevant information. This goal can be achieved by defining the axes of this subspace (called principal components) as those along which the variance of the projected data is maximised, under the constraint of orthogonality. Mathematically, this concept takes the form of the bilinear model, X = TP^T^, where X is the matrix of the original experimental data, T is the matrix containing the coordinates of the samples in the space of the principal components (scores matrix), and P is a matrix describing the contribution of the original experimental variables to the definition of the principal component space (loadings matrix). Chemometric analysis was carried out using the SIMCA (Soft Independent Modeling of Class Analogy, MKS Instruments AB UMEÅ Sweden) program.

## 4. Conclusions

From an overview of the above-reported results, it can be concluded that pH has a remarkable effect on both Sclg and Sclg/borax systems. While in the case of Sclg, flow curves show the stability of the polysaccharide triple helix up to pH = 13 with a breakdown of the viscosity at 9 Pa, in the presence of borax, the initial Newtonian plateau is shifted forward and the viscosity breakdown occurs at approximately 20–30 Pa. On the other side, at pH = 14, for both systems a dramatic decrease of viscosity takes place because of the breakdown of the triple-helix structure together with the chemical degradation of the glycosidic linkages, thus leading to a viscosity that becomes only 4 times higher than that of distilled water. These effects are also clearly evidenced by the swelling behaviour of tablets prepared with the Sclg/borax system. When the tablets were swelled at pH = 14, reduced water uptake and anisotropic elongation were detected due to the beginning of the Sclg chain degradation process, accompanied by the breakdown of the triple helix. When the polymer for tablet preparation was preliminary dissolved at pH = 14, the obtained dosage form showed almost no water uptake and no anisotropic elongation because an almost complete Sclg chain degradation and triple-helix denaturation already occurred during the polymer dissolution (i.e., before the tablet preparation). These effects were reduced in the case of the other two tested polysaccharides, GG and LBG, due to the different initial conformation (coil) and a healing effect (peculiar of these galactomannans) that somehow slow down the chain degradation induced by the high pH value. For a deeper insight about the effect of pH on the structure of the Sclg/borax complex, the interaction between CR and the Sclg triple helix was investigated by spectroscopic and chemometric approaches. The information acquired from these experiments clearly shows that the borax shifts the triple helix denaturation to higher pH values, as evidenced also by the rheological characterisation.

Finally, in agreement with the observed CR-induced CD, MD simulations allowed identification of the most representative structures of the CR–triple-helix complexes: the probe was located mainly along a perpendicular or parallel orientation with respect of the Sclg chain, as already observed in the simulations previously carried out in the absence of borax.

## Figures and Tables

**Table 1:** Linear viscoelastic threshold for the Sclg and Sclg–borax systems (cp = 0.7%). *G*’_c_ and *G*″_c_ are the critical storage and the loss moduli; τ_c_ and γ_c_ are the critical stress and the critical deformation, respectively.

**Scleroglucan**					
**pH**	**5.5**	**10**	**12**	**13**	**14**
***G*’_c_ (Pa)**	12.8	-	14.4	9.7	0.0
***G*″_c_ (Pa)**	4.8	-	4.8	3.6	0.0
**τ_c_ (Pa)**	5.6	-	5.6	4.4	0.0
**γ_c_ (−)**	0.41	-	0.37	0.42	0.0
**Scleroglucan–Borax**					
**pH**	**9.0**	**10**	**12**	**13**	**14**
***G*’_c_ (Pa)**	16.1	14.8	20.0	10.0	0.0
***G*″_c_ (Pa)**	5.1	5.0	5.2	3.7	0.0
**τ_c_ (Pa)**	9.1	7.2	9.1	5.6	0.0
**γ_c_ (−)**	0.54	0.46	0.44	0.52	0.0
